# Performances of Corneal Topography and Tomography in the Diagnosis of Subclinical and Clinical Keratoconus

**DOI:** 10.3389/fmed.2022.904604

**Published:** 2022-05-26

**Authors:** Cristina Ariadna Nicula, Adriana Elena Bulboacă, Dorin Nicula, Ariadna Patricia Nicula, Karin Ursula Horvath, Sorana D. Bolboacă

**Affiliations:** ^1^Department of Ophthalmology, “Iuliu Haţieganu” Medicine and Pharmacy University, Cluj-Napoca, Romania; ^2^Oculens Clinic, Cluj-Napoca, Romania; ^3^Department of Physiopathology, “Iuliu Haţieganu” Medicine and Pharmacy University, Cluj-Napoca, Romania; ^4^Emergency County Hospital, Târgu Mureş, Romania; ^5^Department of Ophthalmology, “George Emil Palade” University of Medicine, Pharmacy, Sciences and Technology, Târgu Mureş, Romania; ^6^Department of Medical Informatics and Biostatistics, “Iuliu Haţieganu” Medicine and Pharmacy University, Cluj-Napoca, Romania

**Keywords:** keratoconus, subclinical keratoconus, corneal topography, corneal tomography, clinical utility

## Abstract

**Aim:**

The purpose of the study was to assess the efficacy of topographical and tomographical indices given by the Pentacam (pachymetric, tomopetric, and aberometric) in clinical and subclinical keratoconus (KCN) diagnosis.

**Material and Methods:**

In this observational analytic retrospective study, patients with abnormal findings in topography and tomography maps but with no signs on clinical examination (subclinical KCN group, sKCN), patients with clinical keratoconus (KCN group), and healthy subjects (Control group) were evaluated.

**Results:**

The KCN group proved significantly different (p < 0.001) values of the investigated parameters than the Control group. Eleven out of 28 investigated parameters proved significantly different in the sKCN group compared to controls (*p* < 0.001). Two topographic measurements, namely I-S (cut-off = 1.435, a large value indicates the presence of KCN) and CCT (cut-off = 537, a small value indicates the presence of KCN), showed AUCs equal to 1 [0.999 to 1]. Six other Pentacam measurements, including Back maximum keratometry (Back Kmax) proved to be excellent parameters for case-finding and screening. In distinguishing sKCN from normal eyes, Pentacam index of vertical asymmetry (IVA), inferior-superior difference (I-S) value, thinnest point (TP), Belin Ambrosio Enhanced Ectasia Display (BAD_D) and root mean square total (RMS total) performed best.

**Conclusions:**

In distinguishing sKCN from normal eyes, Back Kmax, IVA, I-S, and RMS total values demonstrated higher accuracy and utility. Six indices, namely ISV, IVA, KISA, PRC, RMS-HOA, and Back Kmax demonstrate excellent utility in case-finding and screening for clinical KCN.

## Introduction

Keratoconus (KCN) is a progressive corneal ectatic disease that may appear at any age, it is frequently seen in teenagers and dramatically progresses without treatment (1). It is characterized by progressive central thinning of the cornea, irregular astigmatism, and decreased visual acuity. The prevalence of KCN show large variations from population to population, as 0.15% in the United States (2), 4% in the Iranian rural population (3), 37.4 cases (95% CI–confidence interval [36.8 to 37.9]) per 100,000 people in South Korea (4), 192.1 per 100,000 (95% CI [188.3 to 195.9]) in Norway (5). The KCN is more frequently observed in males (OR = 2.30, *P*-value <0.05) (2) and has a family aggregation (3, 6). Index surface variance and index of vertical asymmetry confirm a high association between phenotypes and genetic factors, supporting further investigation of the genetic mechanisms in keratoconus (6). Changes in oxidative stress markers were detected in KCN, indicating that oxidative stress may play a role in the development and disease progression (7). Non-enzymatic antioxidants and decreased expression of genes encoding antioxidative enzymes (aldehyde dehydrogenase, superoxide dismutase, peroxiredoxins, thioredoxin reductase) have been reported as decreased in patients with KCN (8, 9). Eye rubbing, atopy, allergy, asthma, Down syndrome, contact lens wear, myopia and eczema are other risk KCN factors reported in the scientific literature (3, 10, 11). Proteomic studies of tears from keratoconic patients have demonstrated the presence of elevated inflammatory markers, including tumor necrosis factor alfa, interleukin-6, interleukin-17, implicating inflammation in the pathogenesis of KCN (12).

Corneal cross-linking riboflavin-ultraviolet A (UVA) introduced by Wollensak et al. (13) stops the illness's progression, mainly in the early or moderate stages. In the early stages, the visual acuity may be normal and slit-lamp examination cannot give us features of KCN, so corneal topography and tomography re the gold standard examination for diagnosing corneal ectasia. Identifying subclinical keratoconus (sKCN), described by Amsler in 1946 (14), is important in order to assess the candidates for refractive surgery (15). Corneal topography is a non-contact imaging tool that gives information about the anterior surface of the cornea (16). Corneal tomography evaluates anterior and posterior corneal surfaces and is mandatory for preoperatory evaluation before refractive surgery (16, 17). The first signal of an ectatic corneal disease is the posterior elevation (16–20). Most corneal topographical systems are built on Placido disc that analyses rings reflected on the corneal surface, and cannot evaluate the posterior corneal surface. Scheimpflug imaging is the technique used for corneal tomography (17). The Oculus Pentacam® (Oculus Optikgerate GmbH, Wetzlar, Germany) implements the Scheimpflug technology to create topographic reports. The cross-sectional images generated by a rotating Scheimpflug camera are used to locate the anterior and posterior corneal plane (17). The Global Consensus on Keratoconus and Ectatic Disease established in 2015 the following criteria for diagnosing keratoconus: abnormal posterior elevation, abnormal corneal thickness, and corneal thinning (21). The corneal indices offered by Pentacam (I-S index - inferior-superior index, KISA% index - keratoconus percentage index, central K value, AST - astigmatism index, SRAX - steepest radial axes, and BAD_D-Belin/Ambrosio enhanced ectasia display) are used to diagnose the KCN and to establish the disease severity. The KCN staging is concordant with the ABCD grading system proposed in 2016, which is linked with the topographical findings: anterior and posterior curvature within a 3-mm zone centered around the thinnest point of the cornea, thinnest pachymetry, and distance best-corrected visual acuity (22). Rabinowitz (23) reported KISA% index as the best metric for diagnosis (99.6% rate of correct diagnosis). Hashemi et al. (24) showed that index vertical asymmetry (IVA) is the best diagnostic index for KCN, and recommended a combination of indices to obtain accurate results. Bühren et al. (25) reported in a sample fi 17 eyes an AUC (area under the ROC curve) of 0.980, cut-off = −0.2 μm of corneal wavefront (vertical coma) as a diagnosis metric for sKCN. No consent regarding the best cut-off values for diagnosis of KCN was reached (26–32), and multi-parameter evaluation is recommended (33). Furthermore, the cut-off values for diagnosis of sKCN remain to be established. The sKCN term is used to define a very early preclinical stage of the illness in eyes with slight topographic features like clinical KCN, but without the classical keratometry or slit lamp signs (1, 18, 34–36). Previous studies reported some reliable indices from Pentacam to distinguish sKCN from normal eyes (20, 37–40), respectively KCN from normal eyes (14, 37, 38, 41–43).

Several studies revealed the long-term efficacy of standard collagen cross-linking (CXL) (irradiance of 3 mW/cm^2^ for 30 min) in stopping the progression of KCN (44, 45). The accelerated CXL procedure was introduced to reduce treatment time (46–48). Mazzotta et al. (49) showed the effectiveness of 15 mW/cm^2^ pulsed-light epithelium-off accelerated CXL in stabilizing KCN progression. A non-statistically significant intraoperative corneal reduction was also demonstrated in patients undergoing pulsed-light epithelium-off accelerated CXL by using dextran free 0.1% riboflavin solution with hydroxyl-propyl methylcellulose (50). The efficiency of corneal epithelial-disruption CXL in medium-term stabilization of KCN and better comfort for the patient was also reported (51). Improvement of the refraction by corneal reshaping treatments, with a decrease of vertical asymmetry and high order aberrations, is important in treating patients with KCN (52). Selective transepithelial topography guided photorefractive keratectomy combined with accelerated CXL treatment (STARE X protocol) in improving the visual acuity, manifest refraction and high-order aberrations have been reported (53). The placement of intracorneal polymethyl methacrylate segments or rings in the mid-stroma of the peripheral cornea inducing an arc-shortening effect of the corneal geometry that flattens the central area of the corneal tissue is another treatment option that proved efficient (54). Keratoplasty is usually recommended in advanced KCN cases (55).

The scientific literature search has identified no study reporting the Pentacam diagnosis of KCN or sKCN in the Romanian population. Our study aimed to assess the efficacy of topographical and tomographical indices given by the Pentacam (pachymetric, topometric, and aberometric) in clinical and subclinical keratoconus diagnosis and to establish the cut-off values able to discriminate KCN and subclinical KCN from normal corneas.

## Materials and Methods

A retrospective, observational single-center study was performed at the Oculens Clinic in Cluj-Napoca, Romania. According to the Declaration of Helsinki, our study met the bioethical standards and was approved by the Ethical Committee of the Clinic (1/2022).

### Participants

Patients older than 18 years, regardless of gender and with a corneal tomography treated at our clinic between 15 January 2019 and 15 January 2020, were eligible for the study. Patients diagnosed with KCN confirmed by slit-lamp examination (conical protrusion, stromal thinning, Fleisher ring, and Vogt's striae), keratometry and corneal topography, and tomography were included in the KCN group. In our study, KCN was considered as any eye that had an asymmetric bow tie pattern or an abnormal localized steepening, merged with at least one of the following signs: steep keratometry >47 diopter (D), maximum keratometry (Kmax)> 48.7D, oblique cylinder >1.5 D, corneal thickness under 500 μm, abnormal topographical patterns (asymmetric bow-tie), inferior-superior (I-S) difference value>1.9D at 6 mm (3 mm radii), distorted keratometry mires, distortion of the ophthalmoscopic red reflex or clinical KCN in the fellow eye (56, 57). The KCN eyes met the criteria of the Collaborative Longitudinal Evaluation of Keratoconus study (58). The sKCN was considered if the following features were present: no identifiable slit lamp exam, keratometry or ophthalmoscopy signs; asymmetric bow-tie pattern at topography, K max between 47.2–48.7D, I-S values between 1.4D and 1.9D at 6 mm (3 mm radii), abnormal anterior and posterior elevation values, and no history of ocular trauma, ocular surgery or contact lens wear (56, 59).

The control group comprises subjects selected from the candidates for refractive corneal surgery with myopia (< -8.5D) and/or myopic astigmatism (< -3.5D) with stable ocular refraction (unchanged keratometry, spherical equivalent, cylinder) for almost 1 year, and a normal corneal tomography and healthy eyes. No patients with suspect signs of KCN were included among refractive surgery candidates.

Patients with previously intracorneal ring placement, corneal collagen cross-linking, corneal pachymetry <400 μ, concomitant corneal disease, central corneal scar, dry eye syndrome, and ocular eye surgery or trauma were excluded.

### Eyes Evaluation

All eyes had an ocular examination consisting of distance best-corrected visual acuity examination (DCVA), ocular refraction, slit lamp exam, fundus, and intraocular pressure investigation. All patients were requested to discontinue contact lens use for almost 1 month before the corneal topography/tomography. The corneal topography and tomography were performed with the Pentacam® (HR Premium; Oculus Optikgerate GmbH, Wetzlar, Germany) under scotopic conditions, without pupillary dilation, by the same experienced person. All patients had an appropriate position during the examination and were asked to blink a few times before the examination, open both eyes, and gaze at the fixation target. The automatic-release mode started the scan after we obtained a perfect lining up. Twenty-five Scheimpflug images were taken on an optical zone of 9 mm on the cornea on each evaluated eye. For anterior and posterior corneal elevation, a best-fit sphere (BFS) was used as reference surface measurement. The anterior elevation map represents the radial difference between the sphere and the anterior corneal surface. The posterior elevation map is defined as the radial difference between the sphere and the posterior corneal surface. All the elevation maps were displayed with 2.5 μm color-coded scales.

Curved-based parameters, elevation-based data, pachymetric, aberometric, and integrated indices were extracted from the Pentacam software for evaluated eyes, collected, and stored in an Excel database (Table 1).

**Table 1:** Evaluated Pentacam parameters.

**Parameters class**	**Name of the evaluated parameters**
Curved-based	ksteep, kflat, Kmax at the front/back corneal surface, ARC (anterior and posterior average radius of curvature in the 3 mm zone-anterior radial curve), PRC (posterior radial curve), SV (index of surface variance), IVA (index of vertical asymmetry), KI (KCN index), CKI (central KCN index), I-S value, KISA% (KCN index percentage)
Elevation-based	IHA (index of height asymmetry) and IHD (index of height decentration)
Pachymetry indices	TP (thinnest corneal point), PI (progression index), ART max (Ambrosio relational thickness maximum)

We analyzed the Belin/Ambrósio Enhanced Ectasia Display (BAD_D) to establish deviations from normal front and back elevation limits, which represented a meaningful sign of early KCN and was included as an important sign integrated index. Aberometric indices involved the [root mean square total (RMS total)] and the root mean square high order aberration (RMS –HOA).

We used the ABCD staging (22) criteria included in Pentacam to establish the severity scale of KCN (subclinical - sKCN, clinical mild, moderate, advanced).

### Statistical Analysis

The eye was the statistical unit in our study. Participants' sex was reported as number (%) by group and the differences were tested with the Chi-squared test. We reported the measurements as mean±standard deviation or median (Q1 to Q3), where Q is the quartile's value according to the distribution (Kolmogorov-Smirnov test at a significance level of 5%) of data by each group (KCN, sKCN, and control). Comparisons between the three groups were made with the ANOVA test for normally distributed data and Kruskal-Wallis whenever data proved a deviation from the normal distribution. *Posthoc* analysis was applied when ANOVA or Kruskal-Wallis test showed statistically significant results (adjusted significance level of 1.7%).

The analysis of significant differences between groups was further investigated using ROC (receiver operating characteristic). The cut-off value that discriminates between the two groups was established with Youden's index (max(Se+Sp-1), where Se, sensitivity, Sp, specificity). The performances of individual topographical parameters were assessed whenever the AUC (area under the curve) lower bound of the confidence interval showed at least a good accuracy (0.8≤AUC <0.9) (60). The following statistical metrics were used to evaluate performances of the eligible individual topographical parameters: sensitivity (Se, the highest, the better), specificity (Sp, the highest, the better), positive predictive value (PPV, the highest, the better), negative predictive value (NPV, the highest, the better), positive likelihood ratio (PLR>10 indicates convincing diagnostic evidence; 5 < PLR < 10 indicates strong diagnostic evidence), negative likelihood ratio (NLR < 0.10 indicates convincing diagnostic evidence; 0.2 < NLR < 0.1 indicates strong diagnostic evidence), +CUI (positive clinical utility index; +CUI>0.81 indicates an excellent utility in case-findings, 0.64 ≤ +CUI ≤ 0.81 indicates a good utility in case-findings) and -CUI (negative clinical utility index; -CUI>0.

## Results

**Figure 1:**
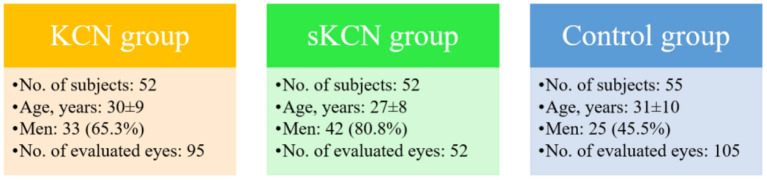
Flowchart with the main characteristics of the evaluated patients. Age is reported as mean ±standard deviation; men are reported as no. and associated percentage.

One hundred and fifty-nine patients aged from 18 to 63 years were evaluated. The number of subjects and the main characteristics are presented in Figure 1. The subjects in the sKCN groups were younger than those in the KCN and Control group, but the difference was not statistically significant (ANOVA test: *P*-value = 0.0632). Significantly more men were in the sKCN and KCN groups than in the Control group (Chi-squared test: *P*-value = 0.0008).

Refractive parameters (sphere, cylinder, spherical equivalent) and distance best-corrected visual acuity of each group are presented in Table 2.

**Table 2:** Refractive parameters and visual acuity of patients in sKCN, KCN and Control group.

**Index**	**Group**			***P*** **-values**			
	**sKCN**	**KCN**	**Control**	**All**	**sKCN vs. KCN**	**sKCN vs. control**	**KCN vs. control**
Sf (D)	−0.8 (−1.3 to −0.3)	−1.5 (−3.3 to −0.6)	−3.8 (−4.8 to −2.8)	<0.0001	0.0099	<0.0001	<0.0001
Cyl (D)	−1 (−1.3 to −0.8)	−2.8 (−4 to −1.8)	−1 (−1.8 to −0.5)	<0.0001	<0.0001	>0.9999	<0.0001
SE (D)	−1.8 (−2.5 to −0.8)	−3 (−4.8 to −1.7)	−4.5 (−5.3 to −3.5)	<0.0001	<0.0001	<0.0001	<0.0001
DCVA (Snellen)	−1 (−1 to −0.8)	−0.4 (−0.7 to −0.3)	−0.7 (−0.8 to −0.5)	<0.0001	<0.0001	<0.0001	0.0005

*D, Diopters; sf, sphere; cyl, cylinder; SE, spherical equivalent; DCVA, distance best- corrected visual acuity. Data are reported as median (Q1 to Q3), where Q1 is the 25^th^ percentile and Q3 is the 75^th^ percentile*.

Topographic and tomographic parameters of all groups from Pentacam are shown in Tables 3, 4. The KCN group proved significantly different values (p <0.001) of the investigated parameters compared to the control group, except corneal volume (VOL) and I-T significant values, compared to sKCN (see Tables 3, 4). Eleven out of 28 investigated parameters proved significantly different in the sKCN group compared with the Control group (Tables 3, 4).

**Table 3:** Pentacam curvature-based parameters by groups: descriptive statistics and comparisons between groups.

**Pentacam parameter**	**Group**			***P*** **-value**			
	**sKCN**	**KCN**	**Control**	**All**	**sKCN vs. KCN**	**sKCN vs. control**	**KCN vs. control**
**Curvature based**							
Front K1 (D)	42.31 ± 0.5	45.73 ± 0.9	42.61 ± 0.7	<0.0001	<0.0001	0.6097	<0.0001
Front K2 (D)	43.9 (42.4 to 44.8)	47.7 (46.3 to 51.6)	44.2 (43.2 to 45.2)	<0.0001	<0.0001	0.8841	<0.0001
Front Kmean (D)	43 (42.1 to 43.8)	46 (44.6 to 49.55)	43.4 (42.6 to 44.6)	<0.0001	<0.0001	0.4313	<0.0001
Front Kmax (D)	45.12 ± 0.5	54.76 ± 0.1	44.61 ± 0.6	<0.0001	<0.0001	0.4395	<0.0001
Rmin	7.50 ± 0.3	6.20 ± 0.6	7.60 ± 0.3	<0.0001	<0.0001	0.3699	<0.0001
Back K1 (D)	−6.1 (−6.2 to −5.98)	−6.5 (−6.9 to −6.2)	−6.1 (−6.2 to −5.9)	<0.0001	<0.0001	>0.9999	<0.0001
Back K2 (D)	−6.4 (−6.7 to −6.28)	−7.1 (−7.55 to −6.8)	−6.5 (−6.6 to −6.3)	<0.0001	<0.0001	>0.9999	<0.0001
Back K max (D)	−6.1 (−6.2 to −6)	−7.2 (−7.5 to −7)	−6.3 (−6.4 to −6.1)	<0.0001	<0.0001	0.0083	<0.0001
ARC (mm)	7.8 (7.5 to 7.8)	6.8 (6.3 to 7.07)	7.8 (7.6 to 7.92)	<0.0001	<0.0001	0.4152	<0.0001
PRC (mm)	6.2 (6 to 6.5)	5.1 (4.6 to 5.36)	6.3 (6.2 to 6.44)	<0.0001	<0.0001	0.8507	<0.0001
ISV	26.21 ± 3	83.63 ± 2	18.55 ± 0.7	<0.0001	<0.0001	0.0300	<0.0001
IVA	0.2 (0.2 to 0.3)	0.9 (0.6 to 1.10)	0.1 (0.1 to 0.13)	<0.0001	<0.0001	<0.0001	<0.0001
KI	1.060 ± 0.05	1.230 ± 0.12	1.010 ± 0.02	<0.0001	<0.0001	0.0012	<0.0001
CKI	1.000 ± 0.02	1.050 ± 0.05	1.010 ± 0.01	<0.0001	<0.0001	0.5257	<0.0001
I-S value (D)	1.2 (0.4 to 1.5)	5.4 (3.7 to 7.83)	0 (−0.4 to 0.35)	<0.0001	<0.0001	<0.0001	<0.0001
I-T value (D)	0.90 ± 0.3	0.80 ± 0.4	0.60 ± 0.2	<0.0001	0.0585	<0.0001	0.0002
KISA (%)	23.42 ± 8.5	11081 ± 584.9	5.38 ± 0.9	<0.0001	<0.0001	0.9127	<0.0001

*Values are reported as mean±(standard deviation) or median (Q1 to Q3) where Q1 is the value of the first quartile and Q3 is the value of the third quartile. The P-value for all subjects is given by ANOVA or Kruskal-Wallis TESTS. The other P-values resulted from the posthoc analysis*.

*Front K1, front keratometry in flat meridian; Front K2, front keratometry in steep meridian; Front Kmean, Front K average; Front Kmax, front maximum keratometry; Back K1, back keratometry in flat meridian; Back K2, back keratometry in steep meridian; Back KmeanBack K average; Back Kmax, back maximum keratometry; R min, minimum sagittal curvature; ARC, anterior radius of curvature in the 3 mm zone; PRC, posterior radius of curvature in the 3 mm zone; ISV, index of surface variance; IVA, index of vertical asymmetry; KI, keratoconus index; CKI, central keratoconus index; I-S, Inferior-Superior index difference; I-T, Inferior temporal index; KISA%, keratoconus percentage index*.

**Table 4:** Pachymetric parameters, elevation based data, integrated indices and aberometric values by groups: descriptive statistics and comparisons between groups.

**Pentacam parameter**	**Group**			***P*** **-value**			
	**sKCN**	**KCN**	**Control**	**All**	**sKCN vs. KCN**	**sKCN vs. control**	**KCN vs. control**
**Pachymetric**							
TP (μm)	521.43 ± 9.4	461.83 ± 4	551.73 ± 1.9	<0.0001	<0.0001	<0.0001	<0.0001
CCT (μm)	529.24 ± 0.4	464.13 ± 1.3	579.44 ± 0.5	<0.0001	<0.0001	<0.0001	<0.0001
ARTmax	339 (271 to 434)	163 (134.5 to 192.5)	458 (417 to 514)	<0.0001	<0.0001	0.0004	<0.0001
IP	1.080 ± 0.25	2.040 ± 0.61	0.990 ± 0.11	<0.0001	<0.0001	0.1726	<0.0001
**Elevation based data**							
IHA	4.7 (2.1 to 12.8)	24.7 (11.7 to 40.85)	4 (2 to 7.6)	<0.0001	<0.0001	0.4704	<0.0001
IHD	0.020 ± 0.02	0.120 ± 0.06	0.010 ± 0.02	<0.0001	<0.0001	0.1540	<0.0001
Cornea vol (mm^3^)	57.8 (55.4 to 62.1)	56.6 (54.7 to 59.4)	61.9 (59.1 to 63.6)	<0.0001	0.2605	<0.0001	<0.0001
**Integrated indices**							
BAD_D	2.1 (1.3 to 3)	7.4 (6.1 to 10.35)	0.9 (0.5 to 1.26)	<0.0001	<0.0001	<0.0001	<0.0001
Q front	−0.4 (−0.5 to −0.3)	−0.7 (−0.9 to −0.5)	−0.4 (−0.5 to −0.31)	<0.0001	<0.0001	>0.9999	<0.0001
Q back	−0.40 ± 0.3	−0.80 ± 0.4	−0.40 ± 0.1	<0.0001	<0.0001	0.3060	<0.0001
**Aberometric**							
RMS Total	31 ± 0.3	193.92 ± 5.8	172.86 ± 0.9	<0.0001	<0.0001	<0.0001	<0.0001
RMS-HOA	0.70 ± 0.4	8.64 ±	1.80 ± 0.3	<0.0001	<0.0001	0.0108	<0.0001
BFS-front	7.940 ± 0.31	7.590 ± 0.42	7.910 ± 0.3	<0.0001	<0.0001	0.6551	<0.0001
BFS-back	6.60 ± 0.22	6.210 ± 0.42	6.430 ± 0.25	<0.0001	<0.0001	0.0023	<0.0001

*Values are reported as mean±(standard deviation) or median (Q1 to Q3) where Q1 is the value of the first quartile and Q3 is the value of the third quartile. The P-value for all subjects is given by ANOVA or Kruskal-Wallis TESTS. The other P-values resulted from the posthoc analysis; TP, thinnest corneal point; CCT, central corneal thickness; ART Max, Maximum Ambrosio relational thickness; IP, pachymetric Index progression; IHA, index of height asymmetry; IHD, index of height decentration; Cornea vol,cornea volume; BAD_D, Belin Ambrosio Enhanced Ectasia Display Total deviation value; Q front, asphericity coefficient front; Q back, asphericity coefficient back; RMS Total,root mean square total; RMS-HOA, root mean square high order; BFS-front, best fit sphere anterior (anterior elevation); BFS-back, best fit sphere posterior (posterior elevation)*.

Excepting KI, I-T, BFS-front and BFS-back, showed AUCs higher than 0.8 regarding discrimination of the KCN by controls (Table 5). Discrimination between subjects suspected by KCN and controls is limited to eighteen topographic measurements, but only I-S, RMS Total, and RMS-HOA showed AUCs excellent accuracy classification (AUC>0.9, Tables 5, 6). Excellent accuracy classification is observed for most investigated topographic parameters when subjects with KCN are compared to those suspected by KCN (Tables 5, 6).

**Table 5:** Curvature based Pentacam parameters AUCs and associated cut-off: KCN vs. controls, sKCN vs. controls, KCN vs. sKCN.

**Pentacam parameter**	**KCN vs. controls**		**sKCN vs. control**		**KCN vs. sKCN**	
	**AUC [95%CI]**	**Cut-off**	**AUC [95%CI]**	**Cut-off**	**AUC [95%CI]**	**Cut-off**
Front K1 (D)	0.783^a^ [0.718 to 0.849]^***^	44.88	0.58 ^b^ [0.483 to 0.676]	42.05	0.821^a^ [0.754 to 0.888]^***^	43.55
Front K2 (D)	0.883^a^ [0.832 to 0.935]^***^	46.05	0.575^b^ [0.478 to 0.671]	42.45	0.896^a^ [0.846 to 0.946]^***^	45.75
Front Kmean (D)	0.845^a^ [0.787 to 0.902]^***^	45.15	0.598 ^b^ [0.503 to 0.693]^*^	42.15	0.873^a^ [0.817 to 0.929]^***^	44.8
Front Kmax (D)	0.981^a^ [0.963 to 0.998]^***^	0.916	0.542^a^ [0.443 to 0.641]	44.35	0.959^a^ [0.927 to 0.99]^***^	48.25
Back K1 (D)	0.782^b^ [0.713 to 0.85]^***^	−6.35	0.525^a^ [0.428 to 0.622]^***^	−6.15	0.789 [0.717 to 0.861]^***^	−6.35
Back K2 (D)	0.884^b^ [0.83 to 0.938]^***^	−6.85	0.55^a^ [0.448 to 0.652]	−6.45	0.875^b^ [0.819 to 0.931]^***^	−6.95
Back Kmax (D)	0.962^b^ [0.931 to 0.993]^***^	−6.75	0.728 ^a^ [0.65 to 0.806]^***^	−6.25	0.974^b^ [0.946 to 1]^***^	−6.35
ISV	0.999^a^ [0.998 to 1]^***^	34.5	0.707^a^ [0.617 to 0.798]^***^	27.5	0.975^a^ [0.949 to 1]^***^	45.5
IVA	1^a^ [0.999 to 1]^***^	0.27	0.857^a^ [0.793 to 0.921]^***^	0.155	0.945^a^ [0.908 to 0.983]^***^	1.105
KI	0.989^a^ [0.972 to 1]^***^	1.065	0.813^a^ [0.735 to 0.89]^***^	1.035	0.829^a^ [0.763 to 0.895]^***^	1.025
CKI	0.835^a^ [0.768 to 0.902]^***^	1.015	0.482^b^ [0.377 to 0.587]	0.995	0.962^a^ [0.931 to 0.993]^***^	0.345
KISA%	0.999^a^ [0.998 to 1]^***^	66.042	0.703^a^ [0.61 to 0.796]^***^	10.35	0.991^a^ [0.981 to 1]^***^	92.322
I-S	1^a^ [1 to 1]^***^	1.435	0.909^a^ [0.864 to 0.954]^***^	0.425	0.979^a^ [0.961 to 0.996]^***^	2.23
I-T	0.662^a^ [0.586 to 0.739]^***^	0.765	0.785 ^a^ [0.702 to 0.867]^***^	0.815	0.612^b^ [0.519 to 0.706]^*^	0.885
Rmin	0.981^b^ [0.964 to 0.998]^***^	7.07	0.534^b^ [0.437 to 0.632]	7.615	0.965^b^ [0.936 to 0.995]^***^	7.075
ARC (mm)	0.97^b^ [0.941 to 0.999]^***^	7.355	0.432^a^ [0.335 to 0.529]	7.615	0.89^b^ [0.814 to 0.965]^***^	7.25
PRC (mm)	0.994^b^ [0.983 to 1]^***^	5.825	0.58^b^ [0.476 to 0.684]	6.225	0.986^b^ [0.971 to 1]^***^	5.7

*Front K1, front keratometry in flat meridian; Front K2, front keratometry in steep meridian; Front Kmean, Front K average; Front Kmax, front maximum keratometry; Back K1, back keratometry in flat meridian; Back K2, back keratometry in steep meridian; Back Kmean,Back K average; Back Kmax, back maximum keratometry; R min, minimum sagittal curvature; ISV, index of surface variance; IVA, index of vertical asymmetry; KI, keratoconus index; CKI, central keratoconus index; KISA%, keratoconus percentage index; I-S, Inferior-Superior index difference; I-T, Inferior temporal index; R min, minimum sagittal curvature; ARC, anterior radius of curvature in the 3 mm zone; PRC, posterior radius of curvature in the 3 mm zone;*

* *p < 0.05;*

^**^0.001 ≤ p < 0.05;

*** *p < 0.001;*

a *larger value indicates stronger evidence for a true positive state;*

b *smaller value indicates stronger evidence for a true positive state*.

**Table 6:** Summary of the pachymetric, elevation, integrated, and aberometric Pentacam parameters: AUCs and associated cut-off for KCN vs. controls, sKCN vs. controls, KCN vs. sKCN.

**Pentacam parameter**	**KCN vs. controls**		**sKCN vs. control**		**KCN vs. sKCN**	
	**AUC [95%CI]**	**Cut-off**	**AUC [95%CI]**	**Cut-off**	**AUC [95%CI]**	**Cut-off**
***Pachymetric***						
TP (μm)	0.98^b^ [0.963 to 0.997]^***^	502	0.716^b^ [0.625 to 0.807]^***^	502	0.871^b^ [0.812 to 0.929]^***^	490.5
CCT (μm)	1^b^ [1 to 1]^***^	537	0.802^b^ [0.719 to 0.886]^***^	543.5	0.898^b^ [0.843 to 0.954]^***^	500.5
ART max	0.983^b^ [0.961 to 1]^***^	345.5	0.786^b^ [0.698 to 0.874]^***^	384.5	0.956^b^ [0.924 to 0.989]^***^	233
IP	0.978^a^ [0.955 to 1]^***^	1.185	0.605^a^ [0.496 to 0.715]^*^	1.145	0.949^a^ [0.915 to 0.982]^***^	1.41
***Elevation based data***						
IHA	0.889^a^ [0.839 to 0.938]^***^	10.3	0.576^a^ [0.475 to 0.677]	10.45	0.83^a^ [0.765 to 0.894]^***^	15.65
IHD	0.984^a^ [0.965 to 1]^***^	0.026	0.747^a^ [0.659 to 0.834]^***^	0.0125	0.972^a^ [0.95 to 0.994]^***^	0.047
Cornea vol (mm^3^)	0.829^b^ [0.771 to 0.887]^***^	57.45	0.743^b^ [0.653 to 0.833]^***^	58.35	0.585^b^ [0.487 to 0.683]	61.2
***Integrated indices***						
BAD_D	0.987^a^ [0.97 to 1]^***^	2.63	0.826^a^ [0.745 to 0.907]^***^	1.52	0.957^a^ [0.919 to 0.995]^***^	4.54
Q front	0.84^b^ [0.782 to 0.899]^***^	−0.515	0.539^a^ [0.441 to 0.637]	−0.465	0.828^b^ [0.759 to 0.897]^***^	−0.485
Q back	0.809^b^ [0.744 to 0.874]^***^	−0.575	0.65^a^ [0.552 to 0.748]^**^	−0.375	0.840^b^ [0.773 to 0.907]^***^	−0.525
***Aberometric***						
RMS Total	0.912^a^ [0.868 to 0.956]^***^	180.842	1^b^ [1 to 1]^***^	149.784	1^a^ [1 to 1]^***^	84.844
RMS- HOA	0.997^a^ [0.992 to 1]^***^	3.011	0.967^b^ [0.94 to 0.993]^***^	1.288	1^a^ [1 to 1]^***^	2.084
BFS front	0.739^b^ [0.67 to 0.808]^***^	7.655	0.587^a^ [0.49 to 0.684]	8.085	0.781^b^ [0.703 to 0.86]^***^	7.85
BFS back	0.67^b^ [0.593 to 0.748]^***^	6.325	0.714^a^ [0.626 to 0.802]^***^	6.515	0.809^b^ [0.74 to 0.878]^***^	6.385

* *p < 0.05;*

** *0.001 ≤ p < 0.05;*

*** *p < 0.001;*

a *larger value indicates stronger evidence for a true positive state;*

b *smaller value indicates stronger evidence for a true positive state; TP, thinnest corneal point;*

*CCT, central corneal thickness; ART Max, Maximum Ambrosio relational thickness; IP, pachymetric Index progression; IHA, index of height asymmetry; IHD, index of height decentration; Cornea vol, cornea volume; BAD_D, Belin Ambrosio Enhanced Ectasia Display Total deviation value; Q front, asphericity coefficient front; Q back, asphericity coefficient back; RMS Total, root mean square total; RMS-HOA, root mean square high order; BFS-front, best fit sphere anterior (anterior elevation); BFS-back, best fit sphere posterior (posterior elevation)*.

According to AUC, refractive parameters and visual acuity show limited diagnosis performances (Table 7). Two topographic measurements, namely I-S (cut-off = 1.435, the large value indicates the presence of KCN) and CCT (cut-off = 537, the small value indicates the presence of KCN), showed AUCs equal to 1 [0.999 to 1] in the differentiation of KCN by controls. All showed the performance metrics of 100% for Se, Sp, PPV, NPP, +CUI and -CUI. Other seven Pentacam measurements, including back Kmax proved excellent for case-finding and screening (Table 8).

**Table 7:** Refractive parameters and visual acuity AUCs and associated cut-off: KCN vs. control, sKCN vs. control, KCN vs. sKCN.

**Parameters**	**KCN vs. control**		**sKCN vs. control**		**KCN vs. sKCN**	
	**AUC [95%CI]**	**Cut-off**	**AUC [95%CI]**	**Cut-off**	**AUC [95%CI]**	**Cut-off**
Sf (D)	0.769^a^ [0.7 to 0.838]^***^	−2.38	0.911^a^ [0.865 to 0.957]^***^	−1.63	0.649^b^ [0.56 to 0.738]^**^	−1.63
SE (D)	0.672^a^ [0.596 to 0.749]^***^	−2.63	0.932^a^ [0.895 to 0.97]^***^	−3.13	0.733^b^ [0.654 to 0.812]^***^	−3.63
DCVA	0.674^a^ [0.598 to 0.75]^***^	−0.45	0.814^b^ [0.744 to 0.884]^***^	−0.75	0.897^a^ [0.845 to 0.949]^***^	−0.65

*p < 0.05;

** *0.001 ≤ p < 0.05;*

*** *p < 0.001;*

a *larger value indicates stronger evidence for a true positive state;*

b *smaller value indicates stronger evidence for a true positive state; Sf, sphere SE-spherical equivalent; DCVA, distance best-corrected visual acuity*.

**Table 8:** Performance analysis of Pentacam indices as diagnosis tools: KCN vs. controls.

**Performance metric**	***ISV***	**Performance metric**	***IVA & KISA & PRC & RMS-HOA***
Se (%)	97.9 [95.0 to 100]	Se (%)	97.9 [95.0 to 100]
Sp (%)	99.0 [97.2 to 100]	Sp (%)	100
PPV (%)	98.9 [96.9 to 100]	PPV (%)	100
NPV (%)	98.1 [95.5 to 100]	NPV (%)	100
PLR	102.79 [14.61 to 723.10]	PLR	n.a.
NLR	0.02 [0.01 to 0.08]	NLR	0.02 [0.01 to 0.08]
+CUI	0.969 [0.938 to 0.999] Excellent for case-finding	+CUI	0.979 [0.954 to 1.000] Excellent for case-finding
–CUI	0.972 [0.952 to 0.991] Excellent for screening	–CUI	0.981 [0.965 to 0.997] Excellent for screening
**Performance metric**	***Back K2***	**Performance metric**	***Back Kmax***
Se (%)	71.6 [62.5 to 80.6]	Se (%)	88.4 [82.0 to 94.9]
Sp (%)	98.1 [95.5 to 100]	Sp (%)	100
PPV (%)	97.1 [93.2 to 100]	PPV (%)	100
NPV (%)	79.2 [72.3 to 86.2]	NPV (%)	90.5 [85.2 to 95.8]
PLR	37.6 [9.47 to 149.15]	PLR	n.a.
NLR	0.29 [0.21 to 0.40]	NLR	0.12 [0.07 to 0.20]
+CUI	0.695 [0.599 to 0.791] Good for case-finding	+CUI	0.884 [0.825 to 0.943] Excellent for case-finding
–CUI	0.777 [0.729 to 0.826] Good for screening	–CUI	0.905 [0.871 to 0.939] Excellent for screening

*n.a., not applicable; Se sensitivity; Sp, specificity; PPV, positive predictive values; NPV, negative predictive value; PLR, positive likelihood ratio; NLR, negative likelihood ratio; CUI, clinical utility index; ISV, Index of surface variance; IVA, index of vertical asymmetry; KISA, keratoconus percentage index; PRC, posterior radius of curvature in the 3.0 mm zone; RMS-HOA, root mean square-high order aberration; Back K2, back keratometry in steep meridian; Back Kmax, back maximum keratometry*.

Six measurements, including back Kmax proved excellent capacities for case-finding and good or excellent capacity for screening in the differentiation of sKNC by KCN, but KISA% showed higher sensitivity, specificity, and clinical specificity utility for case finding and screening (Table 9).

**Table 9:** Performance analysis of Pentacam indices as diagnosis tools: KCN vs. Skcn.

**Performance metric**	***ISV***	**Performance metric**	***IHD***
Se (%)	91.6 [86.0 to 97.2]	Se (%)	92.6 [87.4 to 97.9]
Sp (%)	94.2 [87.9 to 100]	Sp (%)	90.4 [82.4 to 98.4]
PPV (%)	96.7 [93.0 to 100]	PPV (%)	94.6 [90.0 to 99.2]
NPV (%)	86.0 [76.9 to 95.0]	NPV (%)	87.0 [78.1 to 96.0]
PLR	15.87 [5.28 to 47.70]	PLR	9.63 [4.18 to 22.21]
NLR	0.09 [0.05 to 0.17]	NLR	0.08 [0.04 to 0.17]
+CUI	0.885 [0.827 to 0.944] Excellent for case-finding	+CUI	0.877 [0.816 to 0.937] Excellent for case-finding
–CUI	0.810 [0.742 to 0.878] Excellent for screening	–CUI	0.787 [0.713 to 0.860] Good for screening
**Performance metric**	***KISA%***	**Performance metric**	***I-S***
Se (%)	95.8 [91.8 t 99.8]	Se (%)	95.8 [91.8 to 99.8]
Sp (%)	98.1 [94.3 to 100]	Sp (%)	88.5 [79.8 to 97.1]
PPV (%)	98.9 [96.8 to 100]	PPV (%)	93.8 [89.0 to 98.6]
NPV (%)	92.7 [85.9 to 99.6]	NPV (%)	92.0 [84.5 to 99.5]
PLR	49.81 [7.15 to 347.15]	PLR	8.30 [3.91 to 17.64]
NLR	0.04 [0.02 to 0.11]	NLR	0.05 [0.02 to 0.12]
+CUI	0.947 [0.908 to 0.987] Excellent for case-finding	+CUI	0.899 [0.844 to 0.953] Excellent for case-finding
–CUI	0.909 [0.861 to 0.958] Excellent for screening	–CUI	0.814 [0.743 to 0.885] Excellent for screening
**Performance metric**	***PRC***	**Performance metric**	***DCVA***
Se (%)	98.8 [91.8 to 99.8]	Se (%)	72.6 [63.7 to 81.6]
Sp (%)	96.2 [90.9 to 100]	Sp (%)	94.2 [87.9 to 100]
PPV (%)	97.8 [94.9 to 100]	PPV (%)	95.8 [91.2 to 100]
NPV (%)	92.6 [85.6 to 99.6]	NPV (%)	65.3 [54.6 to 76.1]
PLR	24.91 [6.39 to 97.00]	PLR	12.59 [4.17 to 38.03]
NLR	0.04 [0.02 to 0.11]	NLR	0.29 [0.21 to 0.41]
+CUI	0.937 [0.894 to 0.981] Excellent for case-finding	+CUI	0.696 [0.600 to 0.792] Good for case-finding
–CUI	0.890 [0.837 to 0.944] Excellent for screening	–CUI	0.616 [0.534 to 0.698] Fair for screening
**Performance metric**	***Back K2***	**Performance metric**	***Back Kmax***
Se (%)	66.3 [56.8 to 75.8]	Se (%)	95.8 [91.8 to 99.8]
Sp (%)	98.1 [94.3 to 100]	Sp (%)	100
PPV (%)	98.4 [95.4 to 100]	PPV (%)	100
NPV (%)	61.4 [51.0 to 71.9]	NPV (%)	92.9 [86.1 to 99.6]
PLR	34.48 [4.92 to 241.49]	PLR	n. a.
NLR	0.34 [0.26 to 0.46]	NLR	0.04 [0.02 to 0.11]
+CUI	0.653 [0.550 to 0.755] Good for case-finding	+CUI	0.958 [0.922 to 0.994] Excellent for case-finding
–CUI	0.603 [0.524 to 0.681] Fair for screening	–CUI	0.929 [0.886 to 0.971] Excellent for screening

*Se, sensitivity; Sp, specificity; PPV, positive predictive values; NPV, negative predictive value; PLR, positive likelihood ratio; NLR, negative likelihood ratio; CUI, clinical utility index; ISV, index of surface variance; IHD, index of height decentration; KISA, keratoconus percentage index; I-S, inferior-superior index; PRC, posterior radius of curvature in the 3.0 mm zone; DCVA, distance best-corrected visual acuity; Back K2, back keratometry in steep meridian; Back Kmax, back maximum keratometry*.

Only two measurements showed performance in identifying patients with sKCN compared to the controls, but RMS-HOA showed the best performances that support strong diagnostic evidence (Table 10).

**Table 10:** Performance analysis of Pentacam indices as diagnosis tools: sKCN vs. controls.

**Performance metric**	***I-S***	**Performance metric**	***RMS-HOA***
Se (%)	78.8 [67.7 to 89.9]	Se (%)	86.5 [77.3 to 95.8]
Sp (%)	85.7 [79.0 to 92.4]	Sp (%)	99.0 [97.2 to 100]
PPV (%)	73.2 [61.6 to 84.8]	PPV (%)	97.8 [93.6 to 100]
NPV (%)	89.1 [83.0 to 95.2]	NPV (%)	93.7 [89.2 to 98.2]
PLR	5.52 [3.38 to 9.00]	PLR	90.87 [12.88 to 640.96]
NLR	0.25 [0.15 to 0.42]	NLR	0.14 [0.07 to 0.27]
+CUI	0.577 [0.438 to 0.717] Fair for case-finding	+CUI	0.847 [0.755 to 0.939] Excellent for case-finding
–CUI	0.764 [0.708 to 0.820] Good for screening	-CUI	0.928 [0.898 to 0.958] Excellent for screening
**Performance metric**	***sf***	**Performance metric**	***SE***
Se (%)	80.8 [70.1 to 91.5]	Se (%)	100
Sp (%)	91.4 [86.1 to 96.8]	Sp (%)	70.5 [61.8 to 79.2]
PPV (%)	82.4 [71.9 to 92.8]	PPV (%)	62.7 [52.2 to 73.1]
NPV (%)	90.6 [85.0 to 96.1]	NPV (%)	100
PLR	9.42 [4.98 to 17.85]	PLR	3.39 [2.52 to 4.55]
NLR	0.21 [0.12 to 0.37]	NLR	n.a.
+CUI	0.665 [0.535 to 0.795] Good for case finding	+CUI	0.627 [0.513 to 0.740] Fair for case finding
–CUI	0.828 [0.781 to 0.875] Excellent for screening	–CUI	0.705 [0.638 to 0.771] Good for screening

*Se, sensitivity; Sp, specificity; PPV, positive predictive values; NPV, negative predictive value; PLR, positive likelihood ratio; NLR, negative likelihood ratio; CUI, clinical utility index*.

*I-S, inferior-superior difference; RMS-HOA, root mean square-high order aberration; sf, sphere; SE, spherical equivalent*.

## Discussions

Our findings support the usefulness of five Pentacam parameters, ISV (AUC = 0.975), IHD (AUC = 0.972), KISA% (AUC = 0.991), I-S (AUC = 0.979), and PRC (AUC = 0.986), in the differentiation of sKCN from KCN.

De Sanctis (28) reported that posterior corneal elevation measured (100.74 ± 9.2 in KCN and 39.91 ± 5 in sKCN) with the Pentacam is a suitable index for differentiating sKCN from KCN but was less efficient in the diagnosis of sKCN. Nevertheless, a study suggested that an increase in posterior corneal elevation can be an early sign of sKCN (28). Solis-Vivanco et al. (61) reported high specificity (92%) and sensitivity (87%) of corneal topography than the clinical examination. Claude et al. (62) and Rabinowitz (56) support the need to combine different topographical indices to confirm the presence of KCN.

Our results demonstrated that the I-S (AUC = 0.909, cut-off = 0.425, Se = 78.8%, Sp = 85.7%; fair for case-findings and good for screening) and RMS-HOA (Se = 86.5%, Sp=99.0%, AUC=0.967, excellent for case-findings and screening) at the front surface of the cornea were the best variables for the diagnosis of sKCN (Tables 5, 6, 10). Similar results in diagnosing sKCN were reported for I-S by other researchers but with lower performances [AUC = 0.842, Se = 80.1%, Sp = 79.2% (43); AUC = 0.840 (38)]. Hashemi et al. (24) reported that ISV (>0.14) and IVA (>0.22) indices were the best parameters for detecting sKCN cases compared with the well-known indices such as Kmean or K max. Still, our results showed limited performances of these indices and different cut-off values (Table 3). Arbelaez et al. (63) and Heidari et al. (43), using corneal topography, tomography and biomechanical indices, showed that anterior and posterior curvature-based modifications could diagnose sKCN earlier than biomechanical analysis.

In our study, the BAD_D index had an AUC = 0.826 with a cut-off point equal to 1.52 in discriminating sKCN than normal eyes. This cut-off value is like the one reported by Hashemi et al. (24) (1.54), with higher accuracy than the ART Max parameter proposed by Ambrosio et al. (64) in detecting sKCN (cut-off value = 1.45). Correia et al. (65) also supported the idea that BAD_D is an important parameter in diagnosing sKCN and KCN, results also demonstrated in our study (Table 6).

Hashemi et al. (24) reported that BAD_D, IVA, ISV, and 5th order vertical coma aberration are the best criteria for sKCN. Shaag (66) suggested that aberration indices should be simultaneously used with vertical asymmetry.

Our findings revealed that the best indices for discriminating KCN from normal corneas were ISV (AUC = 0.999), ISA (AUC = 0.999, KISA% (AUC = 0.999), PRC (AUC = 0.999), BAD_D (AUC = 0.987), ART Max (AUC = 0.983) and RMS-HOA (AUC = 0.997) (Tables 5, 6). In our study, we took into consideration the ART Max index as a parameter for the diagnosis of KCN with a cut-off value of 345.5 and an AUC = 0.983 (Table 6), a result similar to Muftuoglu et al. (67) that showed high confidence of the ART Max index for the diagnosis of clinical KCN in contrast with sKCN. Muftuoglu et al. (67) introduced the D index as a combination of keratometric, pachymetric, pachymetric progression and back elevation parameters to differentiate KCN from the sKCN. The cut-off value for D index was 1.3, with a sensitivity of 60% and specificity of 90%, suggesting the possible false-negative results (67). In our study, RMS-HOA had a sensitivity of 97.9 and a specificity of 100 (Table 8). Similarly, Hashemi et al. (24) revealed that BAD_D, mean K, and 3rd order vertical coma was optimal for diagnosing clinical KCN. Moreover, Shetty et al. (42) showed that RMS was the best index to distinguish KCN from normal eyes (AUC = 0.983).

Sedgipour et al. (41) showed that KISA% (cut-off value for correct diagnosis of KCN = 100%.) was the single index with specificity and sensitivity >90% demonstrating positive and negative predictive values >95%. Li et al. (36) successfully used K value, I-S value, and KISA% indices to detect KCN.

In our study, corneal volume distribution proved significantly smaller in sKCN group than in controls (*p* < 0.0001, Table 4), supporting its potential in diagnosing sKCN. Similar results were also reported by Ambrosio et al. (29), showing that corneal-thickness spatial profile and corneal volume distribution may differentiate keratoconus corneas from the normal ones.

Our results showed that two topographic measurements, namely I-S (cut-off = 1.435) and CCT (cut-off = 537) were other important parameters in diagnosing KCN. Similarly, Sedhagat et al. (68) demonstrated that I-S value (AUC = 0.986) had the highest capacity among curvature parameters to distinguish KCN from normal eyes. Heidari et al. (43) showed that IHD had better accuracy in diagnosing KCN cases.

Our findings demonstrated the potential of Kmax of the back cornea (Se = 88.4%, Sp = 100%, Table 8) with excellent utility in case-finding and screening of the disease. Similarly, Bae et al. (69) demonstrated that curvature data are more accurate than pachymetric and elevation parameters for early diagnosing KCN. Other studies (70, 71) also reported the usefulness of the posterior corneal surface in the differentiation of the normal eyes from KCN, as the earliest indicator for ectasia. Correia et al. (65) demonstrated that front surface curvature parameters may be applied as objective parameters to diagnose KCN but can be normal in mild ectasia cases. This can give a later ectasia diagnosis than tomographic indices based on posterior elevation and pachymetry distribution (65).

Our study turned its special attention to sKCN cases, proposing effective indices for the diagnosis of this cases, which are applicable and can be evaluated. In our opinion, simultaneous evaluation of BAD_D, back Kmax, IVA, I-S, ART Max and RMS total values can help diagnose cases of sKCN. Even though, these findings do not reduce the importance of the classical indices such as front keratometry. The cut-off points suggested in our study have acceptable specificity and sensitivity. Further studies in this regard would probably conclude in more accurate cut-off values.

Our study has some limitations that need to be highlighted. First, we did not collect the cornea biomechanical parameters, so topographical and tomographical data association was not done. This association could be extremely useful in the early diagnosis of sKCN. Second, based on applied design and collected data, we could not evaluate the sKCN progression to clinical KCN. A prospective cohort study with follow-up evaluation of patients with sKCN will allow the assessment of the rate of progression and is currently conducted by our team. Third, the reported cut-off values must be evaluated on external samples to prove their reliability and validity.

## Conclusions

In distinguishing sKCN from normal eyes, Back Kmax, IVA, I-S and RMS total values were the most accurate indices. All topographical and tomographical parameters had good specificity and sensitivity in diagnosing clinical KCN, but six of them (ISV, IVA, KISA, PRC, RMS-HOA, and Back Kmax) demonstrate excellent utility in case-finding and screening of sKCN. Despite the fact that Back Kmax as a single diagnostic parameter is not sufficient, it does suggest to be very effective in distinguishing sKCN from normal corneas and KCN from normal corneas in association with other parameters.

## Data Availability Statement

The raw data supporting the conclusions of this article will be made available by the authors, without undue reservation.

## Ethics Statement

The study involved human participants and was reviewed and approved by the Ethical Committee of the Oculens Clinic. Written informed consent for participation was not required for this study in accordance with the National Legislation and the Institutional requirements.

## Author Contributions

CN, DN, and AB: study conception and design. CN, DN, KH, and SB: wrote the first draft of the manuscript. AB and AN: data collection. SB: data curation and analysis. All authors contributed to the article and approved the submitted version.

## Conflict of Interest

The authors declare that the research was conducted in the absence of any commercial or financial relationships that could be construed as a potential conflict of interest.

## Publisher's Note

All claims expressed in this article are solely those of the authors and do not necessarily represent those of their affiliated organizations, or those of the publisher, the editors and the reviewers. Any product that may be evaluated in this article, or claim that may be made by its manufacturer, is not guaranteed or endorsed by the publisher.
